# Association of systemic inﬂammation response index with all-cause mortality as well as cardiovascular mortality in patients with chronic kidney disease

**DOI:** 10.3389/fcvm.2024.1363949

**Published:** 2024-02-26

**Authors:** Lu Wei, Shiqing Mao, Xianhong Liu, Chuanqi Zhu

**Affiliations:** ^1^ Department of Respiratory, Mindong Hospital Affiliated to Fujian Medical University, Ningde, China; ^2^ Department of Nephrology, Mindong Hospital Affiliated to Fujian Medical University, Ningde, China

**Keywords:** chronic kidney disease, all-cause mortality, cardiovascular mortality, systemic inflammatory response index, inflammation

## Abstract

**Background:**

Chronic Kidney Disease (CKD) stands as a formidable health challenge, recognized not only for its growing prevalence but also for its association with elevated mortality rates. Emerging evidence suggests that CKD is inherently linked to inflammatory processes, marking it as an inflammatory disorder. In this landscape, the systemic inflammatory response index (SIRI) emerges as a novel inflammation marker, yet to be applied for assessing the risk of mortality in CKD patients.

**Objective:**

This study aims to investigate the prognostic significance of the SIRI in all-cause and cardiovascular disease (CVD) mortality among patients with CKD.

**Method:**

This study conducted a retrospective observational study using the National Health and Nutrition Examination Survey (NHANES) database, encompassing data from 1999 to 2018. This analysis included 9,115 CKD patients, categorized based on SIRI quartiles. Key outcomes were all-cause and CVD mortality, analyzed using Kaplan–Meier survival curves, restricted cube splines (RCS) and cox proportional hazards models.

**Result:**

In this study of 9,115 CKD patients, the Kaplan–Meier survival analysis revealed a greater incidence of all-cause death among groups with higher SIRI (P-log rank <0.001). In the fully adjusted model (Model 3), each unit increase in SIRI led to a 20% increase in the risk of all-cause mortality. Additionally, higher SIRI quartiles (Q3 and Q4) were associated with increased risk compared to the lowest quartile (Q1) (Q3: HR: 1.16, 95% CI: 1.01–1.34; Q4: HR: 1.63, 95% CI: 1.40–1.90; *P* for trend <0.001). Similarly, for CVD mortality, each unit increase in SIRI in Model 3 increased the risk by 33%, with Q3 and Q4 showing higher risk than Q1 (Q3: HR: 1.39, 95% CI: 1.11–1.70; Q4: HR: 2.26, 95% CI: 1.72–2.98; *P* for trend <0.001).

**Conclusion:**

SIRI was positively associated with all-cause and CVD mortality in patients with CKD.

## Introduction

Chronic Kidney Disease (CKD) constitutes a critical health concern, particularly due to its intricate association with heightened mortality rates (1). The implications of CKD-induced mortality extend beyond its primary classification as a renal disorder. Research indicates that CKD affects approximately 13.4% (11.7%–15.1%) of the global population. Meanwhile, a significant number of individuals, estimated to be between 4.902 million and 7.083 million, suffer from end-stage renal disease (ESKD) and require kidney replacement therapy (2). Recent estimates indicate that 1990–2017, the mortality associated with CKD increased significantly by 41.5% (3). Recognizing CKD's role in contributing to the global burden of mortality necessitates a deeper exploration of its pathophysiology and the identification of reliable markers for assessing mortality risk in affected individuals.

Current study recognizes CKD as an inflammatory disorder (4–6). The accumulation of uremic toxins, oxidative stress, and dysregulation of the gut microbiota in CKD patients promote the activation of inflammatory pathways, leading to elevated levels of pro-inflammatory cytokines and other inflammatory markers (7). The intricate interplay of inflammatory processes in CKD progression has become a focal point of research. Inflammation is implicated not only in the initiation but also in the ongoing progression of CKD (8). Therefore, inflammatory markers are expected to be potential biomarkers for assessing the risk of death in patients with CKD.

Amidst the evolving landscape of inflammatory markers, the Systemic Inflammatory Response Index (SIRI) presents itself as a promising candidate. SIRI, calculated as the ratio of neutrophil count multiplied by monocyte count to lymphocyte count. SIRI reflects the balance of host inflammation and immune state, which provides a new perspective for studying the overall inflammatory state in the body (9). Neutrophils and monocytes, elevated in response to inflammation, and lymphocytes, often suppressed, together encapsulate the net inflammatory burden (10). Thus, SIRI reflect the dynamic interplay of pro-inflammatory and anti-inflammatory forces within the body. Zhao et al. indicated that SIRI was associated with increased all-cause and CVD mortality among patients with hypertension (11). Kong et al. indicated Elevated SIRI is associated with an increased risk of all-cause and CVD death in obese people (12). Elevated SIRI has been associated with adverse outcomes in various diseases, including malignancies and cardiovascular diseases (CVD) (13–15). However, its specific role in the context of CKD remains relatively unexplored.

This study aims to bridge existing knowledge gaps by investigating the relationship between SIRI and overall mortality, providing clinicians with a simple yet powerful tool for risk assessment and patient management.

## Methods

### Study population

The study population for this research was drawn from the National Health and Nutrition Examination Survey (NHANES) database, encompassing survey cycles spanning from 1999 to 2018. NHANES, conducted by the Centers for Disease Control and Prevention (CDC) and the National Center for Health Statistics (NCHS) in the United States, is a survey designed to assess the health and nutritional status of the civilian, non-institutionalized population.

**Figure 1:**
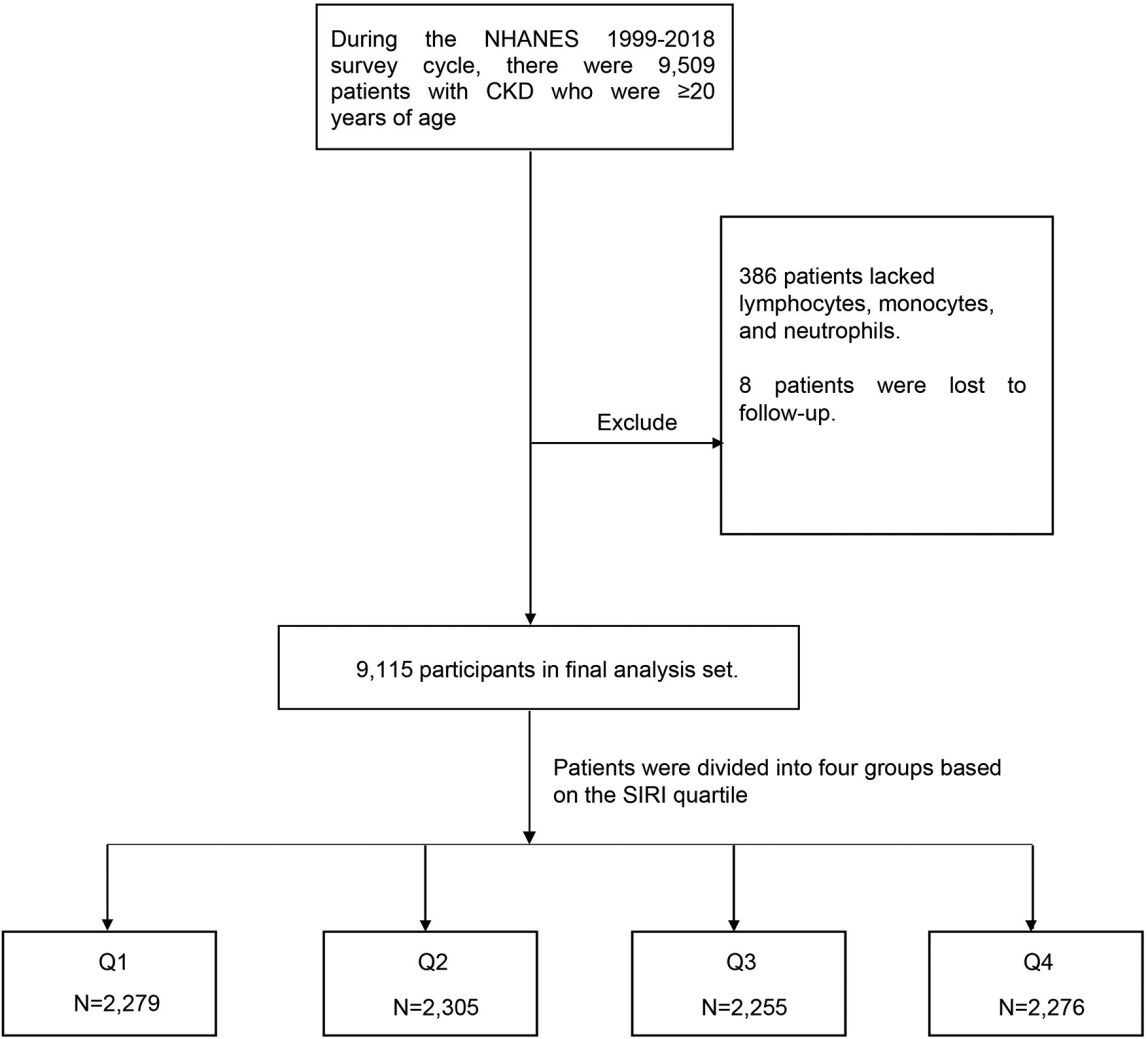
Flowchart of the study design.

The initial dataset comprised a total of 9,509 CKD patients older than 20 years of age identified across the NHANES survey cycles during the specified period. During the data preprocessing phase, certain exclusion criteria were applied. A subset of 386 individuals was excluded due to missing data on essential components required for calculating the SIRI, namely lymphocyte count, monocyte count, and neutrophil count. Additionally, eight participants were excluded due to loss to follow-up. Following the application of these exclusion criteria, a final analytical cohort of 9,115 participants aged 20 and above, with confirmed CKD diagnoses, was established (Figure 1).

### Definition of CKD

Subjects were identified as having CKD if they met any of the following conditions: 1. Estimated glomerular filtration rate (eGFR) less than 60 ml/min/1.73 m^2^; 2. Urinary albumin-to-creatinine ratio (uACR) exceeding 30 mg/g (16).

### Primary outcome

The primary outcome of this research included total mortality and deaths specifically due to cardiovascular disease (CVD). The data was available on the NCHS website (www.cdc.gov/nchs/data-linkage/mortality-public.htm). Death causes were determined using the International Classification of Diseases, 10th Revision (ICD-10). Deaths from cardiovascular causes were identified with ICD-10 codes ranging from I00-I09, I11, I13, and I20-I51, as well as I60-I69 (17, 18). As per the most recent NHANES data update, the follow-up period was extended until 31 December 2019.

### Calculation of the SIRI

SIRI was calculated using the following formula: SIRI = (neutrophil count × monocyte count)/lymphocyte count (15).

### Method of grouping and categorization

The primary analysis involved grouping patients based on quartiles of the SIRI (Q1 group: SIRI ≤ 0.82; Q2 group: 0.82 < SIRI ≤ 1.25; Q3 group: 1.25 < SIRI ≤ 1.88; Q4 group: SIRI > 1.88). To verify the robustness of the results, patients were reclassified in the sensitivity analysis using both the median (1.25) and tertiles (0.96 and 1.61) of SIRI.

### Confounding variable

Self-reporting was used to gather information on age, sex, race, education level, poverty income ratio (PIR), smoking and drinking status, as well as other comorbidities. In addition, patients were also considered to have diabetes mellitus (DM) if they meet one of the following requirements: (1) glycosylated hemoglobin, type A1C (HbA1c) level greater than 6.5%, (2) fasting glucose level equal to or higher than 7.0 mmol/L, (3) random blood glucose level equal to or higher than 11.1 mmol/L, (4) 2-hour oral glucose tolerance test (OGTT) blood glucose level equal to or higher than 11.1 mmol/L, or (5) use of diabetes medication or insulin. Participants who were taking blood pressure medications, or whose systolic or diastolic blood pressure measured by NHANES staff was greater than the threshold (140 mmHg or 90 mmHg) were also considered to have hypertension. Hyperlipidemia is defined as triglycerides ≥150 mg/dl, total cholesterol ≥200 mg/dl, low-density lipoprotein cholesterol (LDL-C) ≥ 130 mg/dl, or high-density lipoprotein cholesterol (HDL) ≤ 40 mg/dl for man, and ≤50 mg/dl for women. In addition, participants who reported using cholesterol-lowering drugs were also defined as having hyperlipidemia. Blood and urine samples were collected by NHANES official staff and sent to professional laboratories for analysis and measurement. Measuring methods in NHANES's official website has a detailed explanation (https://wwwn.cdc.gov/Nchs/Nhanes/).

## Statistical analyses

Since NHANES is a sample survey, all analyses should take into account sample weights. Based on the sampling weight data obtained from the official NHANES website, patients were analyzed using the statistical analysis tutorial provided by NHANES. The representativeness of the sampled samples was illustrated by expressing continuous variables as means (standard error), and categorical variables as a count (weighted percentage). The ANOVA and Kruskal–Wallis test was applied to examine the differences in continuous variables between groups, while the χ^2^ test was performed to assess the differences in categorical variables.

The Kaplan–Meier survival analysis curves and COX proportional risk model were utilized to assess the correlation between SIRI and mortality (including all-cause and CVD mortality). We constructed a grand total of three models. Model 1 was unadjusted, the second model underwent adjustments for age, sex, and race, Model 3 was subjected to adjustments for age, gender, race/ethnicity, smoking status, drinking status, education, PIR, BMI, DM, hypertension, hyperlipidemia and CVD. To visualize the relationship between SIRI and mortality, we plotted a restricted cubic spline (RCS). Subgroup analysis was performed based on age, sex, race, and comorbidities. The multiplicative interaction model was used to explore the interaction between SIRI and stratified variables. Finally, to verify the robustness of the results, we regrouped the patients according to the median and tertiles of SIRI, plotted the Kaplan–Meier survival analysis curves, and performed COX regression analysis as the sensitivity analysis of the study.

All data analyses were performed using the Survey package in R Studio (version 4.2.2). A two-sided *P*-value <0.05 indicated significance for analyses.

## Results

### Baseline feature

This study included a total of 9,115 patients with CKD for analysis. The average age of the patients was 60.9 (0.3) years, with 32.9% having DM, 66.9% having hypertension, 82.1% having hyperlipidemia, and 24.7% having CVD. The patients were divided into four groups (Q1–Q4) based on the quartiles of SIRI. In the group with higher SIRI, the participants had a higher mean age, and there was a gradual increase in the percentage of male patients and smokers. Additionally, a higher prevalence of DM, hypertension, and CVD was observed in the group with higher SIRI. Further details on the baseline characteristics can be found in Table 1.

**Table 1:** Baseline characteristics of the study population (weighted).

Variable	Total (*N* = 9,115)	Q1 (*N* = 2,279)	Q2 (*N* = 2,305)	Q3 (*N* = 2,255)	Q4 (*N* = 2,276)	*P-*value
Age, years old	60.9 (0.3)	56.6 (0.5)	59.2 (0.5)	62.3 (0.5)	65.1 (0.5)	<0.001
Male, *n* (%)	4,315 (42.6)	847 (31.3)	973 (38.0)	1,156 (46.9)	1,339 (53.5)	<0.001
Race, *n* (%)						<0.001
Mexican American	1,367 (6.8)	350 (8.0)	398 (7.2)	355 (7.0)	264 (5.1)	
Non-hispanic black	1,965 (12.0)	838 (22.7)	483 (11.0)	373 (9.1)	271 (6.2)	
Non-hispanic white	4,504 (70.0)	710 (55.0)	1,060 (69.3)	1,232 (73.7)	1,502 (80.8)	
Other hispanic	627 (4.9)	154 (4.8)	191 (5.9)	164 (5.3)	118 (3.4)	
Other race	652 (6.3)	227 (9.5)	173 (6.7)	131 (5.0)	121 (4.5)	
Education level						0.173
<12	3,177 (24.9)	821 (26.9)	827 (23.6)	785 (25.3)	744 (24.2)	
12	2,177 (26.3)	517 (23.8)	540 (27.2)	546 (26.0)	574 (28.1)	
>12	3,739 (48.6)	937 (49.3)	930 (49.2)	917 (48.7)	955 (47.7)	
BMI, (kg/m^2^)	29.9 (0.1)	29.0 (0.2)	30.1 (0.2)	30.1 (0.2)	30.2 (0.2)	<0.001
CRP, (mg/dl)	0.6 (0.0)	0.4 (0.0)	0.5 (0.0)	0.6 (0.0)	1.0 (0.1)	<0.001
Neutrophil, (K/*μ*l)	4.6 (0.0)	3.1 (0.0)	4.1 (0.0)	4.9 (0.0)	6.3 (0.1)	<0.001
Lymphocyte, (K/µl)	2.1 (0.0)	2.6 (0.1)	2.1 (0.0)	2.0 (0.0)	1.7 (0.0)	<0.001
Monocyte, (K/µl)	0.6 (0.0)	0.4 (0.0)	0.5 (0.0)	0.6 (0.0)	0.8 (0.0)	< 0.001
PIR	2.6 (0.0)	2.6 (0.1)	2.7 (0.1)	2.6 (0.1)	2.6 (0.1)	0.054
Smoking, *n* (%)	4,562 (49.7)	984 (40.3)	1,079 (46.8)	1,177 (52.3)	1,322 (59.0)	< 0.001
Drinking, *n* (%)	4,378 (54.0)	1,099 (59.4)	1,101 (61.1)	1,099 (60.3)	1,079 (59.6)	0.797
DM, *n* (%)	3,499 (32.9)	804 (27.2)	861 (31.0)	921 (36.2)	913 (37.4)	< 0.001
Hypertension, *n* (%)	6,532 (66.9)	1,510 (58.1)	1,631 (66.1)	1,642 (68.2)	1,749 (74.5)	< 0.001
Hyperlipidemia, *n* (%)	7,469 (82.1)	1,836 (80.3)	1,883 (81.5)	1,858 (82.9)	1,892 (83.7)	0.100
CVD, *n* (%)	2,557 (24.7)	428 (15.8)	544 (20.1)	678 (26.8)	907 (35.7)	< 0.001
All-cause death, *n* (%)	3,607 (33.6)	657 (24.2)	780 (27.6)	949 (35.4)	1,221 (46.6)	< 0.001
CVD death, *n* (%)	1,098 (10.3)	178 (7.7)	233 (10.4)	302 (14.5)	385 (22.3)	< 0.001

Values are, *n* (%) or mean (SE).

BMI, body mass index; CRP, C-reactive protein; PIR, poverty income ratio; DM, diabetes mellitus; CVD, cardiovascular disease.

### Relationship between SIRI and all-cause mortality

**Figure 2:**
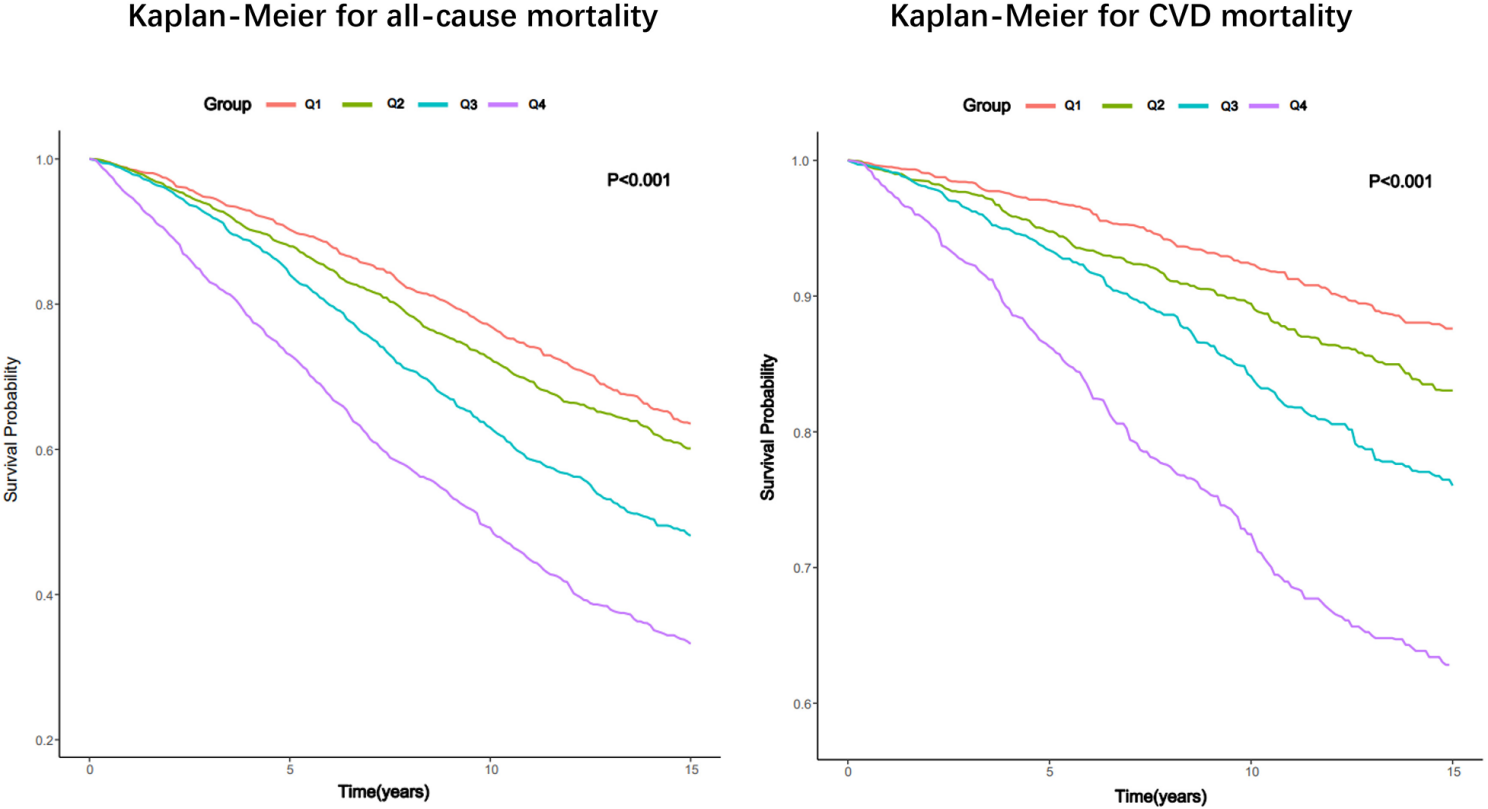
Kaplan–Meier survival estimates for all-cause and CVD mortality in patients with CKD (weighted).

The Kaplan–Meier analysis curves indicated a higher prevalence of overall mortality in group with elevated SIRI (*P*-log rank <0.001, Figure 2). Univariate Cox proportional hazard analysis revealed a 27% elevation in the risk of all-cause death for each unit increase in SIRI. Furthermore, the Q2 [hazard ratio (HR): 1.19, 95% confidence interval (CI): 1.03–1.37], Q3 (HR: 1.64, 95% CI: 1.42–1.89), and Q4 (HR: 2.66, 95% CI: 2.31–3.06) groups exhibited significantly higher risk of all-cause mortality compared to Q1. In Model 3, it was found that every unit increase in SIRI led to a 20% increase in the risk of all-cause mortality. Additionally, Q3 and Q4 groups had a higher risk of all-cause mortality than Q1 (Q3: HR: 1.16, 95% CI: 1.01–1.34; Q4: HR: 1.63, 95% CI: 1.40–1.90; *P* for trend <0.001). The detailed results were presented in Table 2.

**Table 2:** The relationship between SIRI and mortality in patients with CKD (weighted).

Variable	Model 1		Model 2		Model 3	
	HR (95% CI)	*P*-value	HR (95% CI)	*P*-value	HR (95% CI)	*P*-value
All-cause mortality						
Continuous variable						
SIRI	1.27 (1.22–1.32)	<0.001	1.22 (1.18–1.26)	<0.001	1.20 (1.16–1.25)	<0.001
Quartile						
Q1	Ref		Ref		Ref	
Q2	1.19 (1.03–1.37)	0.015	1.11 (0.98–1.27)	0.110	1.02 (0.87–1.18)	0.839
Q3	1.64 (1.42–1.89	<0.001	1.28 (1.14–1.45)	<0.001	1.16 (1.01–1.34)	0.041
Q4	2.66 (2.31–3.06)	<0.001	1.89 (1.67–2.15)	<0.001	1.63 (1.40–1.90)	<0.001
*P* for trend		<0.001		<0.001		<0.001
CVD mortality						
Continuous variable						
SIRI	1.32 (1.25–1.40)	<0.001	1.30 (1.23–1.38)	<0.001	1.33 (1.25–1.41)	<0.001
Quartile						
Q1	Ref		Ref		Ref	
Q2	1.42 (1.10–1.84)	0.008	1.29 (1.02–1.62)	0.032	1.10 (0.85–1.42)	0.462
Q3	2.07 (1.64–2.61)	<0.001	1.58 (1.28–1.95)	<0.001	1.39 (1.11–1.76)	0.041
Q4	3.82 (3.02–4.82)	<0.001	2.63 (2.12–3.27)	<0.001	2.26 (1.72–2.98)	<0.001
*P* for trend		<0.001		<0.001		<0.001

Model 1: Not adjusted.

Model 2: Adjusted by age, gender, race/ethnicity.

Model 3: Adjusted by age, gender, race/ethnicity, smoking status, drinking status, education, PIR, BMI, DM, hypertension, hyperlipidemia and CVD.

### Relationship between SIRI and CVD mortality

Like all-cause mortality, group with elevated SIRI also experienced a greater occurrence of CVD mortality (*P* -log rank <0.001, Figure 2). In unadjusted model (Model 1), the risk of CVD death experienced a 32% surge with each incremental rise of SIRI by 1 unit. And the group displaying an elevated SIRI experienced an increased likelihood of CVD mortality (Q2: HR: 1.42, 95% CI: 1.10–1.84; Q3: HR: 2.07, 95% CI: 1.64–2.61; Q4: HR: 3.82, 95% CI: 3.02–4.82; *P* for trend <0.001). In fully tuned model (Model 3), for every unit increase in SIRI, the risk of CVD death increased by 33%. The group in the third and fourth quartile exhibited a greater propensity for CVD mortality compared to the group in the first quartile (Q3: HR: 1.39, 95% CI: 1.11–1.70; Q4: HR: 2.26, 95% CI: 1.72–2.98; *P* for trend <0.001).

### Regression cubic splines

**Figure 3:**
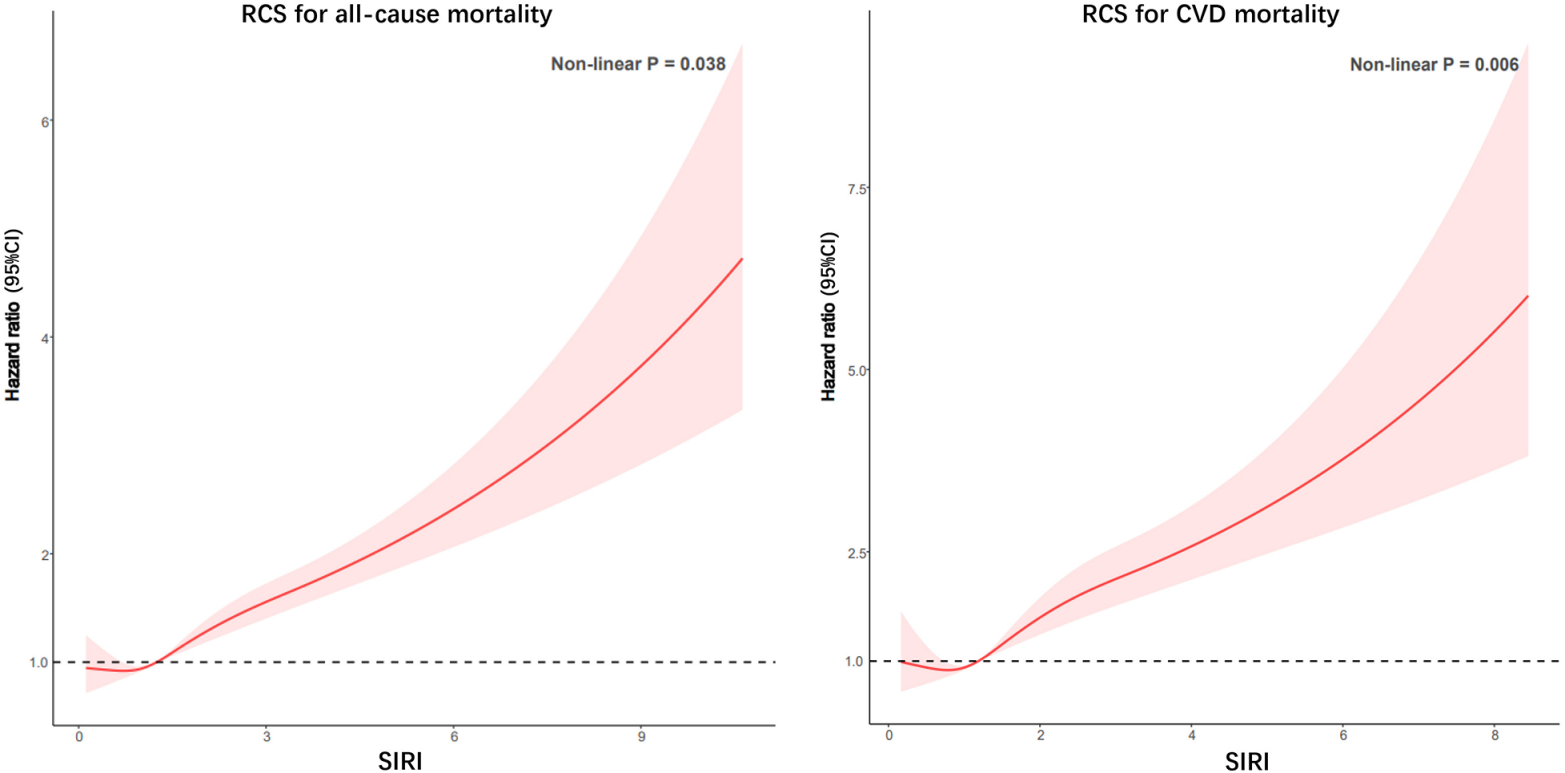
Potential nonlinear relationship between SIRI and all-cause and CVD mortality in patients with CKD (weighted).

After conducting RCS analysis to examine the association between SIRI and both all-cause mortality and CVD mortality, no significant nonlinear relationships were found. The findings suggest that higher SIRI levels are associated with elevated risks of both all-cause death and CVD death (Figure 3).

### Subgroup analysis

**Figure 4:**
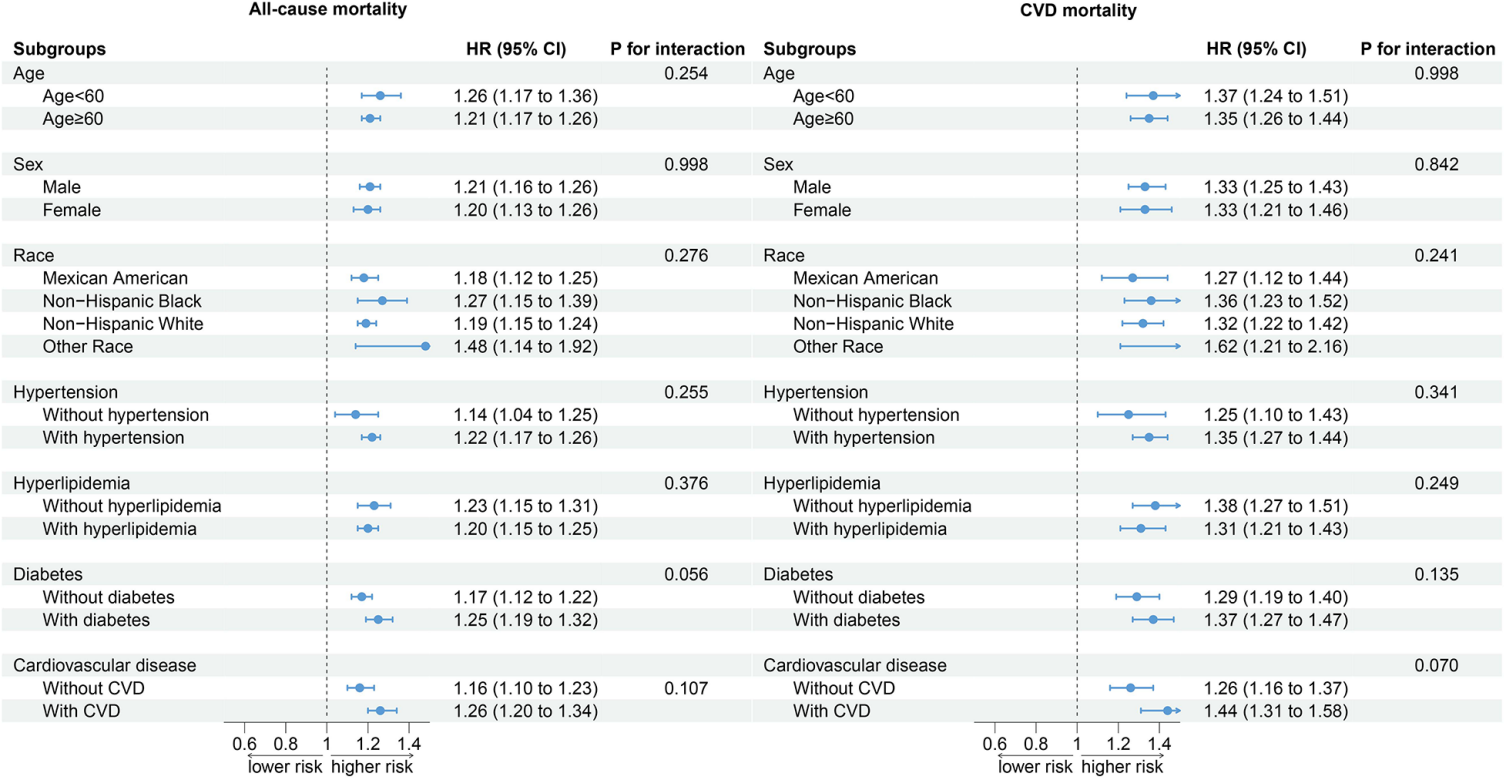
Subgroup analysis.

Stratified analyses based on age, sex, race, hypertension, hyperlipidemia, DM, and CVD showed that the relationship between SIRI and all-cause mortality and CVD mortality in CKD patients was not affected by stratified variables, and no interaction between SIRI and stratified variables was observed (Figure 4).

### Sensitivity analysis

After regrouping patients based on the median and tertile of SIRI. The results of the Kaplan–Meier survival analysis curve and COX proportional risk model show that the relationship between SIRI and mortality (both all-cause mortality and CVD mortality) in CKD patients remains stable (Supplementary Figure S1 and Supplementary Table S1).

## Discussion

In this study, an analysis was conducted on 9,115 patients with CKD, exploring the relationship between the SIRI and all-cause as well as CVD mortality among these patients. The findings revealed that patients with CKD exhibiting higher SIRI values showed a significantly increased risk of both all-cause and CVD mortality. The outcomes remained consistent in sensitivity analysis. These results suggest that SIRI plays a significant role in the mortality risk among patients with CKD.

Mortality rate of CKD patients remains high despite advances in medical care (19, 20). The intricate interplay between inflammation and CKD is pivotal in understanding the elevated mortality risks associated with this condition (4). In CKD, the impairment in renal function leads to an accumulation of uremic toxins, which in turn triggers a systemic inflammatory response. This inflammatory milieu is characterized by the elevation of various cytokines and acute-phase reactants, contributing to the progression of CKD and its complications (21, 22). The persistent state of inflammation in CKD is a critical factor in the development of CVD, the leading cause of death in these patients. Inflammatory cytokines contribute to endothelial dysfunction, atherosclerosis, and myocardial fibrosis. For instance, interleukin-6 (IL-6) and tumor necrosis factor-alpha (TNF-α) have been implicated in the development of atherogenesis and cardiac remodeling (23, 24). Moreover, inflammation synergizes with traditional risk factors for CVD, such as hypertension and DM, further elevating the risk of cardiac events and mortality (25–27).

Our study has found higher SIRI, indicating increased systemic inflammation, was associated with greater mortality (including all-cause and CVD mortality) in CKD patients. This underscores the importance of monitoring SIRI to understand the inflammatory dynamics and their impact on patient outcomes. Several studies have linked inflammatory marker with poor outcomes in CKD. For instance, a study by Ryota Yoshitomi et al. found that neutrophil-lymphocyte ratio (NLR) was predictors of adverse outcomes in CKD patients (28). Kim et al. proposed that the ratio of neutrophils to lymphocytes plays an important role in the prognosis of patients with CKD (29). These studies, along with our findings, suggest a need for a paradigm shift in CKD management, focusing not only on renal function but also on systemic inflammatory status. It is worth mentioning that Zhang et al. has pointed out that despite being easily calculable inflammatory markers, NLR was too simplistic to adequately represent the severity of inflammation (9). Therefore, we chose SIRI covering monocytes to assess the risk of all-cause mortality and CVD mortality in CKD patients.

Monocytes have been recognized as having a role in cardiovascular calcification, which is a leading contributor to mortality in patients with CKD. Monocyt-derived macrophages produce pro-inflammatory cytokines and oxidative stress that contribute to osteogenic transformation and mineralization of blood vessel/valve cells, leading to cardiovascular calcification (30). The results of a research study demonstrated that there exists a correlation between the number of monocytes and the likelihood of developing CKD and the progression of CKD (31). This suggests that monitoring monocyte count could be important for predicting renal outcomes in patients. Some research highlighted that monocyte count modifies the association between CKD and the risk of death (32, 33). These studies indicated that monocytes could be a crucial factor in understanding the mortality risks associated with CKD. These findings collectively underscore the multifaceted role of monocytes in CKD, from predicting the onset of the disease to impacting its progression and associated health outcomes. Therefore, monocytes cannot be ignored when assessing the relationship between inflammation and the risk of death in patients with CKD.

This study utilizes the NHANES database, which represents the population of the United States, thus lending strong extrapolation to the study results. In this study, all three regression models showed that SIRI was strongly associated mortality (including all-cause mortality and CVD mortality) in patients with CKD. Similar results were observed in RCS. In the sensitivity analysis, the result remained unchanged regardless of the way CKD patients were classified. Our study highlights the importance of SIRI as a simple yet powerful tool for assessing mortality risk in CKD patients. The simplicity of calculating SIRI from routine blood tests adds to its practicality in a clinical environment. Unlike other biomarkers that might require specialized testing, SIRI leverages readily available data, making it a cost-effective and easily accessible tool for clinicians. In the future, SIRI is expected to become an important biomarker for clinicians to evaluate the condition and prognosis of patients with CKD.

## Limitations

Our study has certain limitations. The retrospective nature of NHANES data limits our ability to establish causality. Additionally, the reliance on ICD-10 codes for mortality causes might introduce classification bias. Additional longitudinal research is necessary to validate our findings and investigate the mechanisms that contribute to the correlation between SIRI and CKD outcomes. Finally, the patient's blood parameters may vary greatly during a long follow-up, so further research is needed to monitor the patient's SIRI level in the future.

## Conclusions

SIRI was positively associated with all-cause and cardiovascular mortality in patients with CKD. Monitoring SIRI can help identify high-risk patients early, allowing for timely and targeted intervention.

## Data Availability

The raw data supporting the conclusions of this article will be made available by the authors, without undue reservation.
