# Supplementary material for: Association of systemic inﬂammation response index with all-cause mortality as well as cardiovascular mortality in patients with chronic kidney disease
Source: Front Cardiovasc Med. 2024 Feb 26;11:1363949. doi: 10.3389/fcvm.2024.1363949 (PMC10933054; doi:10.3389/fcvm.2024.1363949)
Supplement: Supplementary file 1 [file Table1.docx]

**Supplementary Table 1. The relationship between SIRI and mortality in patients with CKD (weighted)**

| **Variable** | **Model 1** | | **Model 2** | | | **Model 3** | | |
| --- | --- | --- | --- | --- | --- | --- | --- | --- |
|  | **HR (95%CI)** | **P-value** | | **HR (95%CI)** | **P-value** | | **HR (95%CI)** | **P-value** |
|  |  | **All-cause mortality** | | | | |  |  |
| **Upper quartile** |  |  | |  |  | |  |  |
| Group 1 | Ref |  | | Ref |  | | Ref |  |
| Group 2 | 1.91(1.74-2.11) | <0.001 | | 1.47(1.35-1.60) | <0.001 | | 1.36(1.23-1.52) | <0.001 |
| **Tertiles** |  |  | |  |  | |  |  |
| T1 | Ref |  | | Ref |  | | Ref |  |
| T2 | 1.47(1.30-1.67) | <0.001 | | 1.28(1.14-1.44) | <0.001 | | 1.21(1.07-1.38) | 0.003 |
| T3 | 2.41(2.13-2.73) | <0.001 | | 1.77(1.58-1.98) | <0.001 | | 1.58(1.38-1.80) | <0.001 |
| **P for trend** |  | <0.001 | |  | <0.001 | |  | <0.001 |
|  |  | **CVD mortality** | | | | |  |  |
| **Upper quartile** |  |  | |  |  | |  |  |
| Group 1 | Ref |  | | Ref |  | | Ref |  |
| Group 2 | 2.32(1.97-2.74) | <0.001 | | 1.75(1.49,2.05) | <0.001 | | 1.67(1.37-2.04) | <0.001 |
| **Tertiles** |  |  | |  |  | |  |  |
| T1 | Ref |  | | Ref |  | | Ref |  |
| T2 | 1.82(1.48-2.25) | <0.001 | | 1.56(1.28-1.91) | <0.001 | | 1.38(1.11-1.72) | 0.004 |
| T3 | 3.46(2.81-4.26) | <0.001 | | 2.50(2.07-3.03) | <0.001 | | 2.26(1.78-2.86) | <0.001 |
| **P for trend** |  | <0.001 | |  | <0.001 | |  | <0.001 |

Model 1: Not adjusted.

Model 2: Adjusted by age, gender, race/ethnicity.

Model 3: Adjusted by age, gender, race/ethnicity, smoking status, drinking status, education, PIR, BMI, DM, hypertension, hyperlipidemia and CVD.
